# Surgical impact on brain tumor invasion: A physical perspective

**DOI:** 10.1186/1750-1164-2-1

**Published:** 2008-04-02

**Authors:** Thomas S Deisboeck, Caterina Guiot

**Affiliations:** 1 Complex Biosystems Modeling Laboratory, Harvard-MIT (HST) Athinoula A. Martinos Center for Biomedical Imaging, Massachusetts General Hospital, Charlestown, MA 02129, USA; 2 Dept. Neuroscience, University of Torino, Torino, Italy; 3 CNISM, Torino, Italy

## Abstract

It is conventional strategy to treat highly malignant brain tumors initially with cytoreductive surgery followed by adjuvant radio- and chemotherapy. However, in spite of all such efforts, the patients' prognosis remains dismal since residual glioma cells continue to infiltrate adjacent parenchyma and the tumors almost always recur. On the basis of a simple biomechanical conjecture that we have introduced previously, we argue here that by affecting the 'volume-pressure' relationship and minimizing surface tension of the remaining tumor cells, gross total resection may have an *inductive *effect on the invasiveness of the tumor cells left behind. Potential implications for treatment strategies are discussed.

## Background

**Figure 1:**
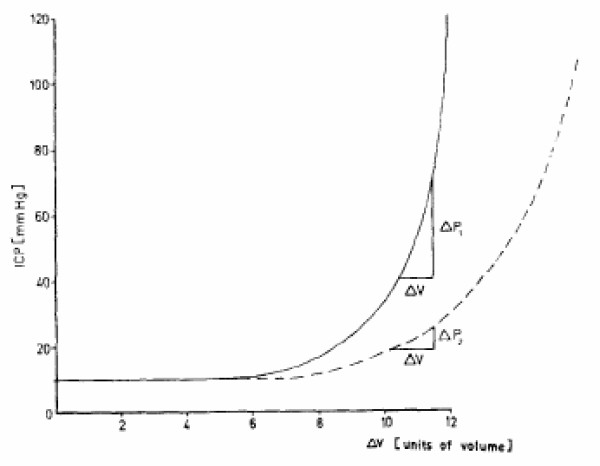
**Intracranial pressure-volume curve**. In the case of lower elastance (*dotted *line), the same increase in volume leads to a lower intracranial pressure increase than in the case of higher elastance (*solid *line) (from Hase et al. [1], with permission).

Malignant brain tumors such as gliomas expand through proliferation and invasion within the confines of the bony skull, hence in a mechanically constraint area. That is, the brain tissue's mechanical 'reserve' rooms, i.e. the interstitial space, the cerebrospinal fluid filled ventricles and the vascular system can only temporarily compensate, through fluid shifts, any sudden or even gradual increase in intracranial pressure (ICP) as it relates to e.g. an intracerebral hemorrhage or a tumor. Once the point of decompensation is reached (Figure 1) any even miniscule increase in volume will trigger a massive, life-threatening increase in ICP [1] that generally leads to surgical intervention in an attempt to resect some of this pressure-raising volume.

Standard treatment for highly aggressive gliomas (anaplastic astrocytomas and glioblastomas (GBM)) has therefore changed little over the years and still consists of gross surgical resection followed by adjuvant radiotherapy and chemotherapy (e.g., [2]). The problem is that the already spatially disseminated tumor cannot be visualized at the single cell level with current clinical imaging modalities, let alone be removed completely. Hence, after a period of temporary betterment, the tumor almost always returns, primarily locally, i.e., at the surgical edge, in conjunction with widespread tissue invasion that at some point prohibits further cytoreductive therapy. Therefore, regardless of any treatment, the grim outcome overall did not change in decades with most GBM patients succumbing to the disease within 1–2 years after diagnosis (e.g., [3]). New approaches are therefore desperately needed and, as always, the development of innovative strategies starts with a better understanding of the current limitations. Here, we discuss, from a pure theoretical and simple mechanistic perspective, if the first line approach of surgical debulking has any impact on the characteristic invasive behaviour of these cancer systems post-extirpation of the tumor core.

## Hypothesis

### Presentation

*In vitro*, invasive gliomas display finger-like invasive patterns in three-dimensional extracellular matrix environments [4], not unlike the chain-like migration seen from gliomas *in vivo *by following axonal fiber tracts and blood vessels [5,6]. Such 'fingering' morphology is common in other physical contexts as well: for instance, **(i) **when a 'crack' occurs in a solid specimen experiencing a point-like internal breakage or, with a growing inclusion inside [7], **(ii) **when a drop of liquid is injected in a more viscous environment ('Hele-Shaw' effect), or **(iii) **when a liquid drop impacts on a solid surface, causing the formation of a fluid 'crown' ('Rayleigh' or 'Yarin-Weiss' capillary instability [8]). Detailed investigations of these models predict the number *N *of fingers which are expected to form depending on the characteristics of the media. Notably, under some very general assumptions, the number of parameters to be accounted for predicting *N *is very low, and can be reduced to the radius *R *of the 'drop' or of the inclusion, the external pressure *P *which acts on it and finally, the surface tension *σ*.

$$N\approx \sqrt{\frac{PR}{\sigma }}$$

As previously proposed in [9], based on **Eq. (1)**, a dimensionless *Invasion Parameter*, *IP, *can be defined assuming that invasion occurs (*IP *> 1) when *N *≥ 1.

*IP *= *PR/σ*

Here, *P *stands for the confining mechanical pressure exerted by the microenvironment on the tumor, *R *for the radius of the tumor, and *σ *for the tension at the tumor surface. According to **Eq. (2)**, invasive cell behaviour is to be expected in all *but *for the case of *IP *< 1 (which implies large tumor surface tension, small confining pressure and/or small tumor radius values).

In the case of malignant gliomas, surgical debulking is geared to reduce the tumor burden, hence *R *should decrease, which in turn would diminish the product *PR *and result in an overall *IP *decline, *if σ *would to remain *constant*. To be more specific, according to Schettini and Walsh [10] the reduction of an intracranial balloon volume from 6 ml to 0.5 ml (i.e., of a factor of 12) would produce a reduction of the product *PR*, being *P *the intracranial pressure measured in the ipsilateral ventricle of a factor ranging from 24 to 8.8 mmHg, i.e., roughly of an order of magnitude. Such measurements, however, are performed 'acutely', i.e., before compensatory mechanisms, due to both the nonlinear viscoelastic behaviour of the brain and circulatory autoregulation, are elicited. Therefore, on a closer look, there are a few important caveats:

• First, this 'volume-pressure' relationship or elastance (and its inverse quantity, compliance) found for the inflated epidural balloon in experimental settings should, in reality, only hold for benign tumors that grow strictly compressive and non-infiltrative in nature such as most meningiomas [11]. In fact, Hase et al. [1] already compared the ICP dynamics post-surgery for such meningiomas versus astrocytomas. The authors reported a higher elastance for meningiomas (comparable to the *solid *line in Figure 1), as compared to a lower elastance for malignant astrocytomas (suggested as *dotted *line; see also **Table 3 **in [1]). That is, in astrocytomas, the same increase in volume leads to a lesser increase in ICP. We argue that the distinct infiltrative nature of these malignant tumors is responsible for causing such 'favourable' mechanical conditions for the neoplasm. (Gliomas for instance release the neutotoxicity-inducing excitatory amino acid glutamate [12] and produce matrix degrading enzymes or metalloproteinases [13] which together can impact and disrupt surrounding tissue architecture, as seen on diffusion tensor NMR [14,15]. None of the complicated biochemical processes driving tumor progression is explicitly included in our hypothesis here; rather, we focus on some simple biomechanical aspects involved). Further, this may delay the onset of purely pressure-related symptoms which in turn can help explain the surprisingly large sizes of some of these malignant tumors at the time of diagnosis. Conversely, surgically reducing tumor volume will also lead to a relatively smaller rate of reduction in *P*. Returning then to **Eq. (2)**, post-surgical intervention, the *PR *product may remain elevated and any sub-total resection (with a sizeable portion of *R *remaining) will only add to this tendency.

• Secondly, by definition, gross total resection will aim to remove the (on conventional imaging, now generally nuclear magnetic resonance, or NMR, imaging) visible surface of the tumor. The Laplace law should hold for the resulting spherical shell and thus, *σ *should, if anything, become very small postoperatively which in turn would yield an excessive increase in *IP *for the remaining, already disseminated cancer cells that remain below the current imaging resolution. It is noteworthy in that context that dexamethasone, a glucocorticosteroid often used peri-operatively with the idea of treating tumoral edema formation, has been shown to also increase the surface tension of the malignant astrocytoma cell lines U-87MG, U-118MG and LN-229 [16]. One could therefore argue that this effect may be one reason why peri-operative steroid therapy is effective to some degree.

• And finally, adding the experimentally proven notion that more invasive cells move faster on stiffer substrates [17-19] to the aforementioned lower elastance of astrocytomas post-extirpation also makes for an invasiveness-promoting microenvironment for the remaining tumor cell formations.

### Implications

In Guiot et al. [9] we had deduced from **Eq. (2) **that increasing *σ *(e.g., through stable E-cadherin transfection as described already for prostate cancer cells in [20]), as well as reducing *P *and *R *are the rather obvious, *distinct *therapeutic goals. While this remains correct, what deserves a more careful evaluation is their *relationship *particularly within the anatomic confines of a host organ such as the brain. Surgical impact, while reducing *R*, temporarily relieving *P *and thus at least subjectively improving the patient's symptoms can, for the reasons stated above, potentially have detrimental *inductive *effects on tumor invasion for the cells left behind, which are generally believed to significantly contribute to recurrence and treatment failure (e.g., [21]). One could therefore argue, that surgery fails to prevent if not even *facilitates *the transition of the tumor from a rather volumetric growth process to a more diffusely expanding system, where infiltrating glioma cells can switch to a proliferative phenotype and vice versa [22]; since such single cell dynamics remain hidden largely below the current NMR imaging threshold, this spatio-temporally propagating system now provides the clinician with lesser if any macroscopic targets to go after, up until a sizeable tumor recurs. However, suggesting not to operate would be irresponsible since the severity of clinical symptoms all too often mandates rapid surgical ICP relief and, not surprisingly, numerous studies have shown that gross total resection improves the patient's disease-free interval (e.g., [23]). That is, aside from obtaining tissue for a histo-pathological confirmation of tumor grade, the main biomechanical goal of any surgical intervention is to approximate the flat part of Figure 1. Our conjecture only cautions about the standard approach's potential side effects on the invasive cells left behind and, in doing so, **(1) **confirms the indication for pharmaceutical peri-operative ICP reduction, and **(2) **reemphasizes the desperate need for effective anti-invasive targeting modalities that are administered *in conjunction *with surgery (and conventional adjuvant regimen). (Surgery of solid malignant tumors usually attempts to excise larger margins in healthy tissue which, however, is a limited option in the brain where any even minimal tissue damage can add to the functional deficit already caused by the tumor).

The former (1) refers also to avoiding an effective increase in *P*, known as an 'ICP rebound phenomenon' that can occur e.g. when the osmotic diuretic agent Mannitol (administered to treat elevated ICP) extravasates into the tissue which in turn can cause a reverse osmotic shift and thus a detrimental raise in ICP [24]. Given that glucocorticoids are still the treatment of choice, one would hope for a renaissance of the pharmaceutical field that is geared towards intracranial pressure reduction. The latter (2) supports for instance further development of the recently introduced anti-angiogenesis compounds. They are designed to impact the nutrient supply system (by targeting integrin receptors [25] or related growth factors such as VEGF [26]), thus supposedly help manage the *PR *dynamics of any residual or recurrent tumor; in doing so, they should also impact structural dissemination routes for invasive glioma cells which are known to move along the perivascular space amongst others [27]. Interestingly, the perivascular space seems to be also a permissive location of cancer stem cells [28] that have been implicated in the development of treatment resistance [29]. Taken together, while recent results raise some caution [30], so far anti-angiogenetic drugs remain a promising strategy as we have argued already in [31] on the basis of organ specific albeit limited carrying capacity.

In summary, following a primarily biomechanical conjecture, the impact caused by undoubtedly necessary surgical intervention may have unintended, inductive effects on invasiveness of the glioma cells left behind. This emphasizes the need for pharmaceutical peri-operative ICP reduction, and supports innovative strategies geared directly towards targeting disseminated cells.

## Competing interests

The author(s) declare that they have no competing interests.

## Authors' contributions

TSD developed the hypothesis and drafted the manuscript, with CG being responsible for the mathematical description. Both authors read and approved the final manuscript.
